# Life, gender, and work: Intersectional data on care work and the living conditions of women in Tumaco, Colombia

**DOI:** 10.1016/j.dib.2025.112171

**Published:** 2025-10-14

**Authors:** Natalia Escobar-Váquiro, Sandra Balanta-Cobo, Lina Buchely-Ibarra, Luis Eslava, Nathalia Maldonado-Polanco

**Affiliations:** a Universidad Icesi, Projects Director – Observatorio para la Equidad de las Mujeres, Colombia; b Universidad Icesi, Researcher – Observatorio para la Equidad de las Mujeres, Colombia; c Universidad Icesi, Director – Observatorio para la Equidad de las Mujeres, Colombia; d La Trobe University, Professor of International Law, Australia; e Universidad Icesi, Research Assistant – Observatorio para la Equidad de las Mujeres, Colombia

**Keywords:** Gender, Work, Care, Tumaco, Survey, Living conditions, Colombia

## Abstract

In this article, we detail a database on the living conditions of women in the ethnically diverse municipality of San Andrés de Tumaco (Tumaco), Colombia. The database was designed and collected by the Observatory for Women's Equity (OEM) – the result of an alliance between Icesi University and WWB Foundation, Colombia – and as part of the Life, Gender, and Work project funded by the universities of Kent, Warwick and Essex (United Kingdom), and Icesi University (Colombia). The collection of data used a feminist economics and an intersectional approach, and adopted an ethnic-territorial focus. The data collected highlights household composition and care work, global/local care chains, subjective well-being, employability, sexual and reproductive rights, perceptions of domestic and care work, and community participation, all vital information for the establishment and functioning of a national system of care in Colombia and pluri-ethnic countries. The information collected on global/local care chains is also the first effort in Latin America to measure migration from the perspective of care work. The sample size is 574 women.

Specifications Table

**Table:** 

Subject	Social science
Specific subject area	Living conditions database for 574 women in Tumaco, Colombia, covering household care, well-being, employability, and community participation.
Type of data	Raw data
Data collection	The survey was conducted in the municipality of Tumaco, and targeted adult women. Due to security concerns, data collection took place at strategic points in the city with high pedestrian traffic. The sampling methodology was designed using quotas based on districts (from 1 to 5) and age groups (18–29 years, 30–39 years, 40–49 years, 50–59 years, and over 60 years).
Data source location	San Andrés de Tumaco, Nariño, Colombia Latitude: 1° 48′ 0′' Norte, Longitude: 78° 45′ 0′' Oeste
Data accessibility	Repository name: Life, Gender, and Work Data identification number: 10.17632/j98d8xbyxb.1 Direct URL to data: https://data.mendeley.com/datasets/j98d8xbyxb/1
Related research article	*‘none’*

## 1 Value of the Data

This research has the potential to increase understanding about women's lives in the racialised population in the Colombian Pacific region. Current understanding is limited due to insufficient data in subnational data collection as a result of the internal armed conflict, and the ethnic-territorial diversity of the region, and the country, as a whole [1,2].

Using a feminist economics approach, and an ethnic-territorial focus, this survey is designed to comprehensively explore intersectionality in relation to care work [3,1,2]. It delves into various intersectional axes such as life cycle, educational level, marital status, care burden, self-identification of ethnicity, disability status, and victim of armed conflict status, providing a deep understanding of women's lives in the Colombian Pacific region, specifically the municipality of San Andrés de Tumaco (Tumaco).

In addition to its ethnic-territorial focus, this survey includes a module on global/local care chains [4,5] and is the first effort in the Latin American region to measure migration from the perspective of care work.

Researchers interested in issues related to women's conditions and everyday experiences, and methodological issues in gender studies, as [3,4,6,7] suggest, can benefit from this data. In particular, data of this type, collected with an ethnic territorial focus, is highly relevant to inform debates on the functioning and establishment of national systems of care in pluri-ethnic and diverse countries like Colombia.

## 2 Background

The motivation behind compiling this dataset stems from the need to understand the living conditions of women in San Andrés de Tumaco, Colombia, an ethnically diverse territory significantly impacted by armed conflict, structural racism, and economic crises. According to the projection for the year 2025 performed by the National Administrative Department of Statistics (DANE), the municipality has a total population of 215,276 people, where 127,601 are located in urban areas (59 %) and 87.675 in rural areas (41 %) [8]. Out of the total population, 49.89 % are men and 50.11 % are women (DANE, 2017). According to DANE’s data (2015),^1^ the municipality contains a total of 9939 Indigenous people, 129,424 Afro-Colombian and mulatto people, 66 Raizal descent, and one person from the Palenque population or from Basilio de Palenque. According to DANE’s data, approximately 63 % of the population is black, mulatto or Afro-Colombian.

Given the scarcity of subnational data in Colombia, particularly in the historically racialised Pacific region, the data collected from the Tumaco region for this dataset aims to fill the gap in the available gender-specific information. The theoretical background of this dataset is rooted in feminist economics, which seeks to make visible the often-undervalued contributions of women through unpaid domestic and care work [9,10]. The methodological framework employs an intersectional approach, considering various axes, such as ethnicity, disability, and victimisation by armed conflict, to provide a comprehensive view of women's experiences.

This dataset is part of the Life/Gender/Work project and was collected by the Observatory for Women's Equity (OEM) – the result of an alliance between Icesi University and the WWB Foundation, Colombia. The Life/Gender/Work project was supported and conducted in collaboration with the University of Kent, the University of Warwick, and the University of Essex in the UK. The survey, encompassing eight modules and 76 questions, was conducted through in-person interviews, ensuring data accuracy despite the challenging context. This dataset stands alone and complements an original research article [1], policy documents and audiovisual materials, by providing empirical evidence to support discussions on gender, work, and care in Tumaco, thereby enriching academic discussions on these critical issues [X]. In particular the information collected in this survey, and the other outputs of the Life/Gender/Work project, is vital for understanding women’s local/global mobility as a result of care work, and for the establishment, and functioning, of a national system of care in Colombia and similar States.

The objective of compiling this dataset is to provide detailed and disaggregated data on the living conditions, care responsibilities, and social realities of women in Tumaco. The survey seeks to highlight the intersectional nature of gender-based inequalities—particularly among Afro-Colombian women—by collecting empirical evidence that informs the design of care policies in ethnically and territorially diverse regions. Furthermore, this dataset aims to contribute to broader academic and policy discussions on the role of unpaid care work, women's mobility, and the structural conditions that shape gendered labor dynamics in the Global South.

## 3 Data Description

The data presented was collected between 22 and 26 November 2021, in the district of Tumaco, which is located in the department of Nariño, on the Colombian Pacific coast. This statistical operation was part of the larger Life/Gender/Work project conducted by the Observatory for Women's Equity and was reviewed by experts in gender and work from the University Icesi, the University of Kent, the University of Warwick, and the University of Essex. The survey questions were associated with various gender-related issues relevant to the Colombian Pacific region.

The unit of analysis consists of adult women (18 years and older, according to Colombian standards), with a sample size of 574 respondents residing in districts 1, 2, 3, 4, and 5 of Tumaco’s urban area. The survey instrument was implemented using a representative random sampling method based on districts and age groups. The OEM’s Measurement Team conducted the questionnaire with an ethnic-territorial and gender focus. Table 1 describes the sample by district and age group.

**Table 1:** Distribution of the sample by age group and commune in Tumaco.

Age groups	Commune^a^						
	1	2	3	4	5	IDK/NA^b^	Total
20–29 years old	72	22	30	6	70	3	203
30–39 years old	24	10	35	7	46	7	129
40–49 years old	23	9	19	5	30	6	92
50–59 years old	21	12	15	3	23	2	76
> 60 years old	19	8	21	4	19	3	74
Total	159	61	120	25	188	21	574

Source: Own elaboration based on data collected.

a *Commune* refers to administrative subdivisions within the urban area of Tumaco.

b IDK/NA = “I don’t know” or “Not applicable”, indicating respondents who did not know or did not report their commune of residence.

The research was conducted in Tumaco due to this municipality’s long history with violence associated to Colombia’s internal armed conflict, structural racism, and endless economic crises experienced in Colombia and similar global South nations. Tumaco is the second most important city in the Colombian Pacific coast, and is part of the Chocó Biogeographic region [9]. Its population is mostly Afro descendants (80 %), with also a presence of indigenous (5 %) and mestizo (33 %) persons. During the past few decades, Tumaco [10] has been the scene of different and acute manifestations of the Colombian armed conflict, with the presence of multiple armed actors, including paramilitary and guerrilla groups, as well as criminal gangs associated with the trade of narcotics. Its condition as a border zone, as a port on the Pacific Ocean, and as a corridor that allows the connection between different departments in Colombia’s south-west regions and Ecuador, fuel armed violence in the municipality [11]. The confluence of armed actors and drug trafficking has made Tumaco an emblematic case of Colombia’s endemic violence, even after the government’s ongoing efforts to negotiate with different armed groups and advance a heavy militarised response to the production and trade of narcotics [12].

In Tumaco, and similar communities, families’ financial income is derived from some formal and informal paid work, as well as unpaid domestic, care and community work, primarily performed by women. These unpaid forms of work are often invisible and undervalued. These jobs, however, are the pillars that create and sustain the immediate, local, and regional social structures within which the Tumaco community exists [13].

The survey includes 76 questions, primarily closed-ended, which allowed for categorical or ordinal coding of responses. Variables were organised thematically into eight modules and analysed through frequency distributions. Age was grouped in standard 10-year brackets; education level according to the highest completed level; and household structure based on typologies used by Colombia’s National Statistical System (SEN). Time-use variables and perception indicators were coded based on pre-established scales or time intervals (e.g., daily hours or agreement levels from 1 to 4). In some cases, response categories were grouped to facilitate analysis and ensure statistical relevance.

Additionally, a limited number of open-ended questions were included to explore emerging dynamics and to complement quantitative insights with contextual and nuanced information. These responses were coded inductively by theme. All variables are presented with their respective categories and valid cases (base = 574). The full survey instrument is available in the supplementary material.

Table 2 summarizes the main sociodemographic characteristics of the surveyed women in Tumaco, including age distribution, education level, marital status, household composition, and time dedicated to unpaid care and domestic work. The majority of women surveyed are between 25 and 34 years old (27 %), and 85.54 % self-identify as Afro-Colombian. Regarding education, 37.8 % have completed high school, while only 8.7 % have university studies. In terms of marital status, 42.66 % of the respondents are single, and 32.17 % live with their partner. In terms of household composition, 41.81 % live in extended families, and 39 % of households have children under the age of five.

**Table 2:** Sociodemographic characteristics of the surveyed women in Tumaco.

Variables	Categories	N	Base (100)
**Age groups**	18 a 24 years old	124	21.6
	25 a 34 years old	155	27
	35 a 44 years old	112	19.5
	45 a 54 years old	80	13.9
	55 a 64 years old	63	10.9
	65 o more years	41	7.2
	Total	574	100
**Education level**	No educational level	91	15.9
	Completed Elementary school	126	21.9
	Completed High school	217	37.8
	Completed technical or technological studies	90	15.7
	Completed University studies	50	8.7
	Total	574	100
**Marital status**	Single	245	42.66
	Has a partner, but does not live with that person	76	13.29
	Has a partner and lives with that person	185	32.17
	You are married	32	5.59
	You are divorced	19	3.32
	Widowed	17	2.97
	Total	574	100
**Type of household**	One-person family	41	7.14
	Nuclear family without children	27	4.7
	Nuclear family with children	161	28.05
	One-parent family	90	15.68
	Extended family	240	41.81
	Compound family	14	2.44
	Multi-family household	1	0.17
	Total	574	100
**% Of households with children under five years of age in the home**			224
**% Of households with elderly, dependent or disabled people who require attention and care at home**			110
**Number of children**	0	134	23.33
	1	115	20
	2	125	21.75
	3	90	15.61
	More than three	111	19.31
	Total	574	100
**Time dedicated to unpaid care and domestic work**	Nothing	22	3.88
	Less than an hour	65	11.29
	One to four hours	304	52.91
	Four to eight hours	93	16.23
	More than eight hours	90	15.7
	Total	574	100
**% Of women in paid employment**			309
**% Of women who are currently studying**			115
**Ethnic self-recognition**	Afro-Colombian	491	85.54
	Indigenous	7	1.22
	Mestiza	52	9.06
	White person	5	0.87
	Other	4	0.7
	None	15	2.61
	Total	574	100
**Victim of armed conflict**	Forced displacement	354	61.6
	Dispossession of land	14	2.5
	Forced abandonment of land	11	1.9
	None of the above	194	33.8
	Total	574	100
**% Of people with permanent disability to work**			88

Source: Own elaboration based on data collected.

The time dedicated to unpaid domestic work is significant: 52.91 % of the women spend between one and four hours a day on these tasks. Additionally, 61.6 % of the women have been victims of forced displacement, reflecting the impact of the armed conflict on women in the region. On the other hand, 53.8 % of the respondents have some form of (formal and informal) paid employment, and 20 % are currently studying, showing a relatively high participation in both labour and educational activities.

## 4 Experimental Design, Materials and Methods

The dataset is based on primary data collected through a structured survey applied to 574 women in Tumaco, Colombia, between November 22 and 26, 2021. The survey was designed and implemented by the research team from the Observatory for Women’s Equity (OEM) at Universidad Icesi, following feminist economics and intersectional methodological frameworks. The questionnaire covered eight thematic modules: sociodemographic characteristics; global/local care chains; subjective well-being; employment and income; sexual and reproductive health; perceptions of domestic and care work; community participation; and housing conditions.

The sampling strategy was probabilistic, stratified, and representative, with quotas established by communes and age groups, ensuring territorial diversity within the municipality. Data collection was carried out in three zones of the city, chosen for their high pedestrian traffic and territorial relevance.

Given Tumaco’s geographical location and the municipality’s security concerns, this survey was conducted in person at strategic public locations. The selection of these sites aimed to ensure both safety and accessibility for participants. The survey duration averaged around 14 min per respondent. Enumerators received specific training and used digital forms to conduct the interviews. Informed consent was obtained from all participants, and data anonymization, cleaning, and processing were carried out by the OEM team.

The seven survey modules were developed, in addition to the socio-economic and household characterisation module, to capture women’s living conditions, occupation, and care work. The data collected from the survey was subsequently processed and analysed using appropriate statistical methods to derive insights into women's living conditions and experiences in Tumaco.

It is important to underline that this data collection effort makes a unique contribution to the understanding and documentation of women’s mobility associated to care work, offering valuable insights from a global South context on this important issue.

Each table presents three columns: (1) the *variable* measured; (2) the corresponding *response categories* or groupings; and (3) the percentage over the total number of valid responses (*base = 574*, unless otherwise specified). This structure allows for a clear and consistent reading of the survey results across modules.

**Global/local care chains:** This module consists of various categories of questions related to experiences of travelling abroad for work, the type of work performed, the duration of the trip, caring for children or elderly individuals, financial assistance sent from abroad, and plans for travelling to work. It also asks whether the respondent cares for someone at home because the primary caregiver is working abroad and to what extent household expenses can be covered with financial assistance from abroad. Finally, it inquires as to whether the respondent would accept a job offer in another country.

Table 3 summarizes the patterns of labor migration and the configuration of global and local care chains among women in Tumaco, highlighting their work-related mobility, care responsibilities during migration, and the composition of their support networks. From the data collected, it is revealed that about 33 % of women intend to migrate to another region or country to work, and 27 % have already done so. Most of these labour migrations occurred between one and five years ago (42.7 %), with stays typically lasting between three months and one year (30.8 %). The main types of work performed during these migrations include household work (31.1 %), general services (20.3 %), and food preparation (18.9 %). Additionally, 54 % of the women had children at the time of travel, with 54.3 % taking their children with them, while 45.7 % left them in the care of others, primarily their mothers (40.5 %) and grandmothers (21.6 %).

**Table 3:** Global and local care chains: labor migration, care responsibilities, and support networks of women in Tumaco.

Variables	Categories	Base (100)
**Current intention to travel to another country or region for work**	Yes	33
**National or international migratory experience to work**	Yes	27
**How long ago was the last time you travelled for this reason?**	Less than one year ago	17.3
	More than one year ago but less than five years ago	42.7
	More than five years ago	40
**Length of stay**	Less than three months	26
	Between three months and one year	30.8
	Between one year and five years	28.1
	More than five years	15.1
**Main work performed**	Household work	31.1
	General services or housekeeping	20.3
	Food preparation	18.9
	Others	14.9
	Care of children or teenagers	11.5
	Sales	10.8
**Having a child(ren) at the time of travel**	Yes	54
**Childcare while you travelled**	They travelled with you	54.3
	Left them in someone else's care	45.7
**Relationship of your children's primary caregiver**	Mother	40.5
	Grandma	21.6
	Sister	13.5
	Father	10.8
	Another female relative	8.1
	Aunt	5.4
	Brother	2.7
	Another male relative	2.7
	An unfamiliar woman	2.7

Source: Own elaboration based on data collected.

*Note:* Percentages refer to respondents who reported migration experiences or intentions, and may not add to 100 % due to multiple responses.

**Subjective well-being:** This module includes variables related to health, concerns about food availability in the household, perceptions of poverty, current and comparative living conditions, overall life satisfaction, and perception of changes in living conditions in Tumaco over the past five years. Subjective well-being is understood as a concept encompassing people's assessments of their lives, and it has been a concept incorporated into national surveys to complement other objective measures of well-being [14].

The *subjective well-being* module included perception-based indicators measured through single-response questions using categorical scales. These variables included self-assessment of health (Very good to Bad), self-perception of poverty (Yes/No), and comparative assessments (e.g., current vs. childhood economic condition).

Participants were asked about their living conditions compared to that of the past five years, allowing for an assessment of changes in Tumaco. This process provides valuable insights into the perceived improvement or deterioration in various aspects of life, such as infrastructure, public services, economic opportunities, and social well-being, thereby enhancing our understanding of women’s situations.

By examining subjective well-being, this module contributes to a comprehensive understanding of the women's experiences and perspectives on their quality of life, and the socio-economic conditions in Tumaco. It provides valuable insights into the subjective dimensions of well-being beyond traditional objective indicators, allowing for a more nuanced analysis of the impact of living conditions on their overall satisfaction and perception of their lives.

Table 4 presents women’s perceptions of their living conditions and subjective well-being in Tumaco, including self-rated health, economic conditions, and satisfaction with life. Only 4.6 % of respondents rate their health as very good, while the majority (47.1 %) consider it good. A significant 75.6 % worried about running out of food in the past 30 days, and 72.2 % consider themselves poor. Regarding current living conditions, 66 % describe their living conditions as regular, though 51.3 % believe their household standard of living has improved over the last five years. Additionally, 58.9 % view their current economic situation as better compared to their childhood. In terms of life satisfaction, 67.3 % report being satisfied, although 21.3 % express dissatisfaction. Lastly, when asked about the living conditions in Tumaco over the past five years, 48.4 % said they have remained unchanged, while 35.8 % feel they have worsened.

**Table 4:** Subjective well-being and perceptions of life conditions among women in Tumaco.

Variables	Categories	(100)
**Do you consider that your general state of health is…?**	Very Good	4.6
	Good	47.1
	Regular	41.8
	Bad	6.5
**In the last 30 days, at any time, were you worried about food running out in your household?**		75.6
**Do you consider yourself poor?**		72.2
**Currently the living conditions in your home are:**	Very Good	2.7
	Good	30.0
	Regular	66.0
	Bad	1.4
**Do you consider that the standard of living of your current household compared to what it was 5 years ago is…?**	Same	27.6
	Better	51.3
	Worse	21.1
**Do you consider that the economic conditions with respect to your childhood home are…?**	Same	20.6
	Better	58.9
	Worse	20.5
**How satisfied or dissatisfied do you feel with your life in general?**	Very satisfied	9.4
	Satisfied	67.3
	Dissatisfied	21.3
	Very dissatisfied	1.9
**Do you consider that the living conditions in Tumaco in the last 5 years are…?**	Better	15.8
	Worse	35.8
	Remained unchanged	48.4

Source: Own elaboration based on data collected.

**Productive employment/income:** This module of questions focuses on the respondents occupation, unpaid domestic and care work, savings for old age, and received subsidies. The questions include the time spent on domestic and care tasks, occupational category, time and remuneration, actions taken to secure financial stability in old age, subsidies received in the past year, and current educational status.

The aim of this module is to gather data on the employment situation and income-related aspects of women in Tumaco. It captures information about their occupational status, including the type of work they engage in, the time devoted to work, and the level of remuneration. Additionally, it explores the time women dedicate to unpaid domestic and care work, shedding light on the additional responsibilities they bear.

The module also addresses women's preparations for old age by examining their savings and strategies for financial security in the later stages of life. It includes questions about received subsidies, which provide insights into the support systems available to women in Tumaco.

By incorporating these questions, the survey seeks to understand the dynamics of productive employment, income generation, and financial planning among women in the community. This information is crucial for comprehending the economic well-being and the specific challenges faced by women in Tumaco, and similar municipalities in Colombia’s Pacific Coast, and the country more generally. Importantly, it enables policymakers and organisations to develop targeted interventions and support systems to address women’s needs, including national systems of care that responds to women’s present and future needs.

Table 5 describes women’s participation in productive employment in Tumaco, their income levels, and the main financial strategies they use to sustain their households and prepare for old age. Over half (52.9 %) dedicate between one and four hours daily to unpaid care and domestic work, while 15.7 % spend more than eight hours. Regarding subsidies, 25.8 % receive ``Familias en Acción'' (a conditional cash transfer programme established in Colombia in 2021, designed to provide financial assistance to low-income families while promoting childhood education and health), and 19.6 % benefit from aid for displaced persons. In terms of preparing for their old age, 61.8 % do not have any saving system, while 16 % save for the future. About 54 % of respondents engage in economic activities, with 19.5 % employed by public or private entities, and 23.5 % involved in other types of work. Most work between four and eight hours a day (48.2 %), and 35.7 % earn between 200,000 and 500,000 COP monthly (between USD$45 to USD$115 in 2024 terms).

**Table 5:** Productive employment, income, and financial strategies among women in Tumaco.

Variables	Categories	Base (100)
**Hours per day dedicated to housework and/or caring for people**	Nothing	3.9
	Less than an hour	11.3
	One to four hours	52.9
	Four to eight hours	16.2
	More than eight hours	15.7
**Subsidies received**	Unemployment benefits	0.9
	“Jóvenes en acción”	5.1
	“Colombia mayor”	7.9
	VAT refund	14.4
	“Ingreso solidario”	15.3
	Aid for displaced	19.6
	“Familias en Acción”	25.8
	None	40.4
**Activities preparing for old age**	Pay insurance on your own	3.3
	Contribute to a voluntary pension fund	4.2
	Contribute to a mandatory pension fund	9.5
	You prepare your children so that they can support you in your old age	14.4
	Saves	16
	Nothing	61.8
**Do you currently carry out any economic activity?**	Yes	54.0
**Economic activities**	Fisherwoman	1.3
	Cultivator	2.0
	Do household activities in another home	8.4
	Help in the home business or in that of a relative	12.1
	Prepares food and sells it on the street, or sells merchandise, or food	14.1
	You are employed in a trade such as stores, bars, cafeterias, hairdressers	19.1
	You are employed by a public or private entity	19.5
	Other job	23.5
**Dedication time**	Four to eight hours	48.2
	More than eight hours	35.5
	One to four hours	16.4
**Monthly remuneration**	Work free	3.7
	<200.000$ COP	30.3
	Between 200.000$ and 500.000$ COP	35.7
	Between 500.000$ and 900.000$ COP	17.5
	Between 900.000$ and 1.800.000$ COP	9.4
	>1.800.000$ COP	3.4

Source: Own elaboration based on data collected.

*Note:* COP = Colombian Pesos. Percentages refer to the full or a relevant subset of the sample.

**Sexual and reproductive rights:** This module addresses several variables related to the sexual and reproductive health of the survey respondents. Some of the variables include the age of the partner during the first sexual relationship, frequency of cervical screenings, desire to have children, current contraceptive method used, main reasons for not using any contraceptive method, partner's opinion on contraceptive use, items used during the menstrual period, and economic difficulties associated with their purchase. It also asks whether the person has had to suspend or interrupt their work or study due to their menstrual period.

This module aims to gather information on the sexual and reproductive rights of women surveyed in Tumaco. It explores aspects related to their sexual life, such as the age of the partner during the first sexual relationship and the frequency of cervical screenings to understand their access to, and attention to, sexual health.

The desire to have children and the current use of contraceptive methods are investigated, along with the main reasons for not using any contraceptive method. Additionally, the module inquires about the partner's opinion on contraceptive use, providing insights into communication dynamics and decision-making in partner relationships.

The module also addresses aspects related to menstruation, such as the items used during the menstrual period and the economic difficulties associated with their purchase. It asks whether women have had to suspend or interrupt their work or study activities due to their menstrual period, allowing for an understanding of potential barriers to their full participation in different areas of life due to menstrual issues.

This focus on sexual and reproductive rights aims to collect valuable data on the health and well-being of women in Tumaco, as well as identify potential areas for improvement in sexual and reproductive health care and access. This data can be used to inform policies and programs that promote equality and the full exercise of sexual and reproductive rights for women in the community.

Table 6 presents information on sexual and reproductive health among women in Tumaco, including contraceptive use, cervical screening practices, fertility intentions, and menstrual care habits. About 31 % of women used a contraceptive method during their first sexual intercourse, and 40.2 % currently use one. Regarding cervical screening, 33.1 % had one less than a year ago, while 16.4 % have never had one. In terms of family planning, 68.4 % do not wish to have more children, and 66.7 % of those with partners report that their partner agrees with contraceptive use. Menstrual health data show that 91.1 % use disposable sanitary towels, with 31 % experiencing financial difficulties in obtaining menstrual products. Additionally, 20 % have had to suspend work or study due to menstrual periods.

**Table 6:** Sexual and reproductive health and menstrual care among women in Tumaco.

Variables	Categories	Base (100)
**Did you use any contraceptive method during your first sexual intercourse?**	Yes	31
**How long ago was the last cervical screening done?**	Less than one year ago	33.1
	One to three years	34.8
	More than three years ago	15.6
	Never	16.4
**Desire to have children or more children**	Yes, but you want to wait two years or more	8.3
	Yes, and I would like it to be in the next two years.	23.3
	No	68.4
**Are you currently using any contraceptive method?**	Yes	40.2
	No	59.8
**Does your partner agree that you use a contraceptive method?**	Yes	66.7
	No	7.6
	I do not have partner	25.8
**Have you menstruated in the last year?**	Yes	78
**Principal elements used during the menstrual period**	Disposable sanitary towel	91.1
	Tampon	10.6
	Reusable sanitary towel	2.4
	Absorbent Underwear	2.4
	Menstrual cup	0.9
**Experiencing financial difficulties to buy the necessary items to attend to their menstrual period.**	Yes	31
**Suspension of usual activities, work, or study because of menstrual periods**	Yes	20

Source: Own elaboration based on data collected.

*Note:* Percentages refer to respondents who answered each specific question. Some questions were asked only to subgroups (e.g., women with partners or menstruating women).

**Perceptions about domestic and care work:** This module examines individuals' perceptions regarding domestic and caregiving work and the distribution of gender roles within the family and society. The first question focuses on whether the person has enough time for leisure and rest activities. The following three questions use a 1 to 4 scale to measure the person's level of agreement or disagreement with statements related to domestic and care work, and gender roles within this context.

The first question asks for the person's opinion on whether women are better suited for domestic work than men. The second question focuses on the person's perception of whether a mother who works outside the home can be as good of a mother as one who exclusively devotes herself to household chores. The third question centres around the person's perception of whether a woman's main goal should be to get married and have children.

By exploring these perceptions, the module aims to shed light on attitudes and beliefs regarding domestic and caregiving responsibilities and traditional gender roles. The data collected provides valuable insights into community expectations of gender roles, contributing to a better understanding of the social context surrounding domestic and care work, and potentially leading to positive changes in these areas.

Table 7 summarizes women’s perceptions of gender roles and domestic work in Tumaco, including their agreement with traditional gender norms and their sense of time available for personal and leisure activities. About 65.8 % of the respondents feel they have enough time for restful activities like sleeping, exercising, or hobbies. Regarding the statement, ``Women are better at housework than men,'' 46 % strongly agree, while 5.2 % strongly disagree. When asked whether a working mother is as good as one who only does housework, 45 % strongly agree, and only 1.8 % strongly disagree. Finally, in response to the statement ``A woman's main goal is to get married and have children,'' 52.2 % strongly disagree, while 9.8 % strongly agree.

**Table 7:** Perceptions of gender roles and domestic work among women in Tumaco.

Variables	Categories	(100)
You feel that you have enough time for restful activities such as sleeping, exercising, crafts, going to the hairdresser, or playing activities		65.8
On a scale of 1 to 4, (1 being strongly disagree and 4 strongly agree), how much do you agree with the following statement: ``*Women are better at housework than men*''?	1	5.2
	2	14.0
	3	34.8
	4	46.0
On a scale of 1 to 4, how much do you agree or disagree with the following statement: ``*A mother who works outside the home is as good a mother as one who only does housework*''?	1	1.8
	2	15.2
	3	37.9
	4	45.0
On a scale of 1 to 4, how much do you agree or disagree with the following statement: ``*A woman's main goal is to get married and have children*''?	1	52.2
	2	26.6
	3	11.4
	4	9.8

Source: Own elaboration based on data collected.

*Note:* Response scale from 1 (Strongly disagree) to 4 (Strongly agree). Percentages reflect individual responses to attitudinal statements on gender and care roles.

**Community participation:** The questions in this module focus on social participation and problem-solving within the community. The questions are divided into several categories, including involvement in organisations and groups, the type of participation, the methods used to solve problems, the issues that motivate problem-resolution, and the categories of organisations or groups in which the respondents have participated in the past year. Some categories of organisations and groups include community action boards, collectives promoting the rights of ethnic groups, women's rights, work cooperatives or unions, and churches or religious groups, among others. The questions also explore the issues that motivate individuals to be involved in their communities and seek solutions, such as discrimination, violence, environmental protection, corruption, and insecurity.

By examining community participation, the module aims to understand the level of engagement and involvement of individuals in their local community. It provides insights into the types of organisations and groups that individuals are involved in and the issues that drive their participation. This data can be a beacon of hope, identifying areas where community participation can be strengthened and harnessed for positive social change and development in Tumaco. In particular it highlights the women’s community work in addition to their care responsibilities.

Table 8 presents data on women’s participation in community organizations, their problem-solving strategies, and the main motivations that drive collective action in Tumaco. While 67.2 % of respondents did not participate in any organisations, 17.4 % were involved with religious groups, and 11.8 % participated in community action councils. Among those who were active, 42.2 % attended as participants, while 33.9 % were involved in decision-making with voice and vote. In addressing community issues, 11.3 % participated in protests or demonstrations, and 10.5 % held meetings with their community. The most common issues that motivates action were the community and/or domestic violence (28.4 %), corruption (25.6 %), and discrimination (20.9 %).

**Table 8:** Community participation, problem-solving practices, and motivations among women in Tumaco.

Variables	Categories	(100)
**Have you participated in any of these organisations and/or groups in the past year?**	Community action councils	11.8
	Collectives promoting the rights of ethnic groups	4.0
	Collectives that promote women's rights	5.1
	Educational groups	4.9
	Worker cooperative or labour union	1.6
	Churches or religious groups	17.4
	Recreational, sports, artistic and/or cultural associations or groups.	6.1
	Political parties and / or movements	2.1
	Environmental or Animalist Group	3.0
	None	67.2
**What kind of involvement have you mainly had?**	I just be a participant	42.2
	You have participated in the execution of specific activities or tasks delegated by others.	23.9
	You have participated in the decisions with voice and vote	33.9
**To solve some type of problem that affects your community or affects you in the last year, you:**	Used media such as television, radio or print media.	4.9
	Asked for help from civic leader or political leader.	7.5
	Participated in protests, demonstrations, or public marches	11.3
	Held meetings and collective work with members of your community.	10.5
	Sent messages through social networks such as Facebook, Twitter, Instagram, etc.	9.1
	None	73.3
**What issues motivated you to solve the problems that affect you or your community?**	Discrimination (sexual, ethnic and gender)	20.9
	Rejection of violence	28.4
	Defence and protection of the environment	21.1
	Corruption	25.6
	Increased insecurity	19.5
	Violation of Human Rights	24.7
	Rejection of policies and non-compliance with plans and programs proposed by those in power.	16.7
	Tariffs, quality in the provision or lack of public services	12.4
	To require compliance with the right to education, health or labour guarantees.	12.7
	Mobility, transportation or road problems in your municipality or department.	100.0
	Increase in the amount of property tax to be paid	100.0
	None	53.8

Source: Own elaboration based on data collected.

While this article is intended to provide structured, accessible, and well-documented data without interpretations or conclusions—as per Data in Brief’s editorial guidelines—this dataset has been developed as part of a broader research initiative titled *Life, Gender, and Work: Intersectional Data on Care Work and the Living Conditions of Women in Tumaco, Colombia*, led by the Gender Equity Observatory at Universidad Icesi. The findings presented here have informed complementary analyses on care systems, economic inequality, and public policy, which are publicly available through the project’s platform (https://www.icesi.edu.co/oem/proyecto-vida-genero-trabajo) and detailed in the working document *Tumaco: trabajo de cuidado, economía y género* [15]. Researchers and decision-makers are encouraged to use this data for further studies and to inform evidence-based policy interventions in similar contexts.

## Limitations

The dataset described in this article has several limitations. First, security concerns in Tumaco, due to the presence of illegal armed groups in the region, restricted the areas where data collection could take place safely. This may have led to the underrepresentation of certain regions or demographics within the municipality. Additionally, the geographic and infrastructure challenges of the Colombian Pacific region further complicated data collection, potentially impacting the comprehensiveness of the dataset.

The reliance on in-person interviews, while valuable for accuracy, also introduced potential biases, as respondents might have been influenced by social desirability bias or the interviewer’s presence. Though careful sampling methodology, which was designed and based on quotas for districts within the municipality and age groups to reflect the diversity within these groups, was employed, doubt can be had regarding the practical achievement of this aim.

Furthermore, the dataset primarily focuses on women residing in Tumaco’s urban areas, potentially overlooking women's experiences in rural areas. The region's cultural context and low mathematical literacy could also affect the quality and reliability of the responses. Despite these limitations, the dataset provides crucial insights into women's lives in a region where such data is scarce*.*

## Ethics Statement

The ethics committee of the Icesi University has classified the research of the Observatory for Women's Equity as low risk. All procedures are in accordance with the code of ethics of the Icesi University and the respondents gave their consent for the use of the information in accordance with Law 1581 of 2012 on data protection.

## Credit Author Statement

**Natalia Escobar-Váquiro:** Conceptualisation, methodology, validation, formal analysis, investigation, resources, data curation, writing (original draft), review and editing, visualisation, supervision, project administration. **Sandra Balanta-Cobo**: Conceptualisation, methodology, validation, formal analysis, investigation, resources, data curation, writing (original draft), review and editing, visualisation, supervision. **Lina Buchely-Ibarra**: Conceptualisation, methodology, validation, investigation, resources, writing (original draft), review and editing, visualisation, funding acquisition. **Luis Eslava**: review and editing, visualisation, supervision, project **administration**, funding acquisition. **Natalia Maldonado:** Data curation, writing (original draft), visualisation.

## Data Availability

Mendeley DataLife, Gender, and Work: Statistical Data to Understand the Living Conditions of Women in San Andrés de Tumaco, Colombia (Original data).

## Footnotes

1 Cited by the Tumaco’s Local Development Plan (2016 – 2019).
